# Profiling of Small Molecular Metabolites in *Nostoc flagelliforme* during Periodic Desiccation

**DOI:** 10.3390/md17050298

**Published:** 2019-05-18

**Authors:** Xiang Gao, Bin Liu, Boyang Ji

**Affiliations:** 1 School of Food and Biological Engineering, Shaanxi University of Science & Technology, Xi’an 710021, China; 2 School of Life Sciences, Central China Normal University, Wuhan 430079, China; ccnuliubin@gmail.com; 3 Department of Biology and Biological Engineering, Chalmers University of Technology, 41296 Gothenburg, Sweden

**Keywords:** cyanobacteria, *Nostoc flagelliforme*, metabolic profiling, LC-MS, rehydration and dehydration, metabolites

## Abstract

The mass spectrometry-based metabolomics approach has become a powerful tool for the quantitative analysis of small-molecule metabolites in biological samples. *Nostoc flagelliforme*, an edible cyanobacterium with herbal value, serves as an unexploited bioresource for small molecules. In natural environments, *N. flagelliforme* undergoes repeated cycles of rehydration and dehydration, which are interrupted by either long- or short-term dormancy. In this study, we performed an untargeted metabolite profiling of *N. flagelliforme* samples at three physiological states: Dormant (S1), physiologically fully recovered after rehydration (S2), and physiologically partially inhibited following dehydration (S3). Significant metabolome differences were identified based on the OPLS-DA (orthogonal projections to latent structures discriminant analysis) model. In total, 183 differential metabolites (95 up-regulated; 88 down-regulated) were found during the rehydration process (S2 vs. S1), and 130 (seven up-regulated; 123 down-regulated) during the dehydration process (S3 vs. S2). Thus, it seemed that the metabolites’ biosynthesis mainly took place in the rehydration process while the degradation or possible conversion occurred in the dehydration process. In addition, lipid profile differences were particularly prominent, implying profound membrane phase changes during the rehydration–dehydration cycle. In general, this study expands our understanding of the metabolite dynamics in *N. flagelliforme* and provides biotechnological clues for achieving the efficient production of those metabolites with medical potential.

## 1. Introduction

Low molecular weight metabolites, often called small molecules, are important components of the cellular metabolism of a cell, tissue, organ, or entire organism [1,2]. Primary metabolites meet the needs of physiological activities and serve as precursors for the synthesis of secondary metabolites. Secondary metabolites are nonessential for the normal growth of an organism but empower their survival upon interspecies competition and the defensive capability against abiotic stresses or serve as bioactive compounds for medical applications [3]. Mass spectrometry-based metabolic profiling is a powerful tool for the detection of small molecules in cyanobacteria [1]. It employs gas chromatography or liquid chromatography to separate small molecules and mass spectrometry or nuclear magnetic resonance to identify them [4]. Metabolomic analyses can be categorized as either nontargeted or targeted. The former is a nonbiased quantitative analysis of lots of small molecules found in a biological sample while the latter analyzes a specific group of small molecules [1]. Metabolomic analyses, with the output of either molecular dynamics or novel molecules, allow a better and more comprehensive understanding of cellular metabolic processes and their flexibility under diverse environmental conditions in organisms [1,2].

Cyanobacteria are photoautotrophic bacteria capable of oxygenic photosynthesis, which are distributed in diverse ecosystems ranging from temperate terrestrial, freshwater, and marine to extreme environments [5,6]. They are rich sources of bioactive molecules and protective pigments. Various bioactive metabolites have been identified in cyanobacteria, such as cyanotoxin, siderophores, phytohormones, sunscreen pigments, algicides, protease inhibitors, immunosuppressants, and antimicrobial and anticancer compounds [7,8,9]. They can be structurally classified as polyketides, amides, alkaloids, fatty acids, indoles, lipopeptides, etc. [9]. In marine cyanobacteria, an important driving force for producing diverse metabolites is supposed to prevent intense competition or defend predators [9]. In terrestrial xeric environments, cyanobacteria must withstand repeated rehydration–rehydration cycles or periodic desiccation. They are capable of surviving complete dehydration (also called “anhydrobiosis”) and fairly soon resume metabolic activities when they are rehydrated with liquid water [10]. Abiotic stresses will trigger changes in gene expression and protein production, both of which are amplified at the metabolome level [1]. Thus, another important driving force for metabolite production should be the need for coping with abiotic stresses, especially for terrestrial cyanobacteria. Therefore, deciphering the metabolic dynamics as well as the production of novel metabolites in cyanobacteria adapting to xeric environments will help to answer fundamental questions about desiccation tolerance.

*Nostoc flagelliforme,* an edible anhydrobiotic cyanobacterium, is distributed in the xeric steppes of the west and northwestern parts of China [11]. It is present as a filamentous colony form consisting of numerous trichomes surrounded by dense exopolysaccharide. The Chinese have used it as a food delicacy for about 2000 years, and its herbal value has been recognized since 400 years ago [11,12]. Recently, proteomics analyses have revealed the dynamic changes of *N. flagelliforme* proteomes in response to rehydration and dehydration processes [13,14]. However, metabolomic insights into both processes are still lacking. In the present study, we conducted an untargeted liquid chromatography–mass spectrometry (LC-MS) analysis of small molecules in *N. flagelliforme* in response to the rehydration and dehydration processes. The acquaintance of small molecule signatures would extend our understandings on the environmental adaptation of *N. flagelliforme* and might also provide clues for its biotechnological exploitation for food or medical purposes.

## 2. Results and Discussion

### 2.1. Overview of the Rehydration and Dehydration Processes

**Figure 1:**
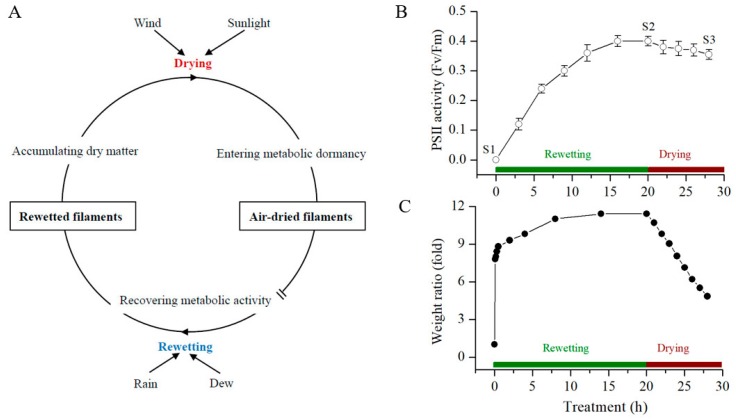
The overview of *N. flagelliforme* in response to the rewetting–drying cycle. (**A**) a descriptive model for native environmental adaptation. (**B**) the changes in PSII activity (in terms of Fv/Fm), and (**C**) the relative water contents of the samples, respectively, during the rewetting and drying processes. The data shown in (**B**) are means ± S.D. (*n* = 5). S1, S2, and S3—three sampling points.

The filaments of *N. flagelliforme* undergo frequent dehydration–rehydration cycles in native environments [15]. *N. flagelliforme* remains in a metabolically inactive state under desiccation and soon resuscitates and enters an active state when it is rehydrated (Figure 1A). The physiological activities of *N. flagelliforme* recover in the order of respiration, photosynthesis, and nitrogen fixation within several hours after being rewet under the light [11]. The photosystem II (PSII) activity parameter Fv/Fm (the ratio of variable fluorescence to maximal fluorescence) serves as a reliable indicator for the changes of physiological states in *N. flagelliforme* during the rehydration or dehydration process [16,17]. As shown in Figure 1B,C, the water contents of the desiccated samples (S1) were rapidly increased at the onset of rewetting, and their Fv/Fm values showed a gradual increase during the rehydration process. The stable maximal Fv/Fm values of the samples were achieved at 20 h (S2) after the rewetting. Following subsequent air-drying for 8 h (S3), ~50% of water in the fully rehydrated samples was lost, and the Fv/Fm values were slightly but significantly (*t*-test, *p*-value < 0.05) reduced. Samples at the three stages (S1, S2, and S3) were collected for metabolite profiling, which represent three different physiological states, respectively: Dormant, physiologically recovered, and in drying.

### 2.2. LC-MS-Based Metabolomic Analysis

**Figure 2:**
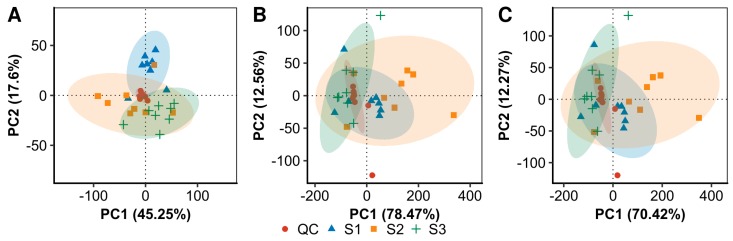
The PCA analysis of the LS-MS metabolomic profiles in positive mode (**A**), negative mode (**B**), and the total ion chromatograms (**C**). The three groups of samples from the three time points (S1, S2, and S3) and pooled QC samples were shown.

To explore the metabolome dynamics during the rehydration–dehydration cycle, we conducted an untargeted metabolic profiling of the above-collected *N. flagelliforme* samples. A total of 2047 features in positive mode and 1697 features in negative mode were detected, of which 1324 and 1041 unique metabolites were respectively confirmed with 165 of them detected in both modes. Principal component analysis (PCA) based on the features from positive and negative modes was employed to compare the metabolic compositions among the samples (Figure 2). The two principal components in positive mode explained 62.9% of the overall variance of the metabolite profiles, with 45.3% and 17.6% for PC1 and PC2, respectively. The two principal components in negative mode explained 91.1% of the overall variance of the metabolite profiles, with 78.5% and 12.6% for PC1 and PC2, respectively. For the total ion chromatograms, the two principal components explained 82.7% of the overall variance, with 70.4% and 12.3% for PC1 and PC2, respectively. Quality control (QC) samples (a mixture of all samples in equal proportions) were clustered together, and the dispersion of the QC samples was obviously lower than the other three groups of samples, which indicated stable measurements and no significant batch bias in sampling. In addition, the PCA analysis revealed distinct metabolomes among the three groups of samples either in positive or negative mode.

### 2.3. Metabolic Signatures in the Rehydration and Dehydration Processes

**Figure 3:**
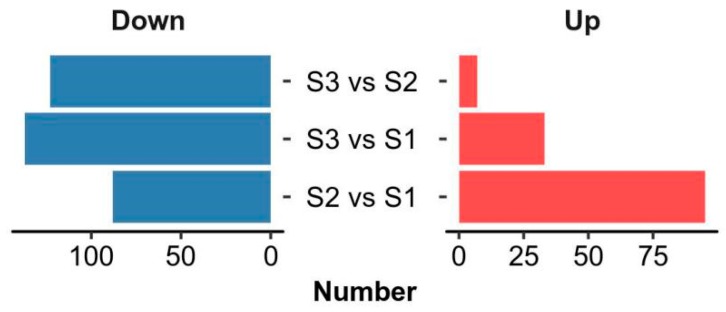
A summary of the differentially regulated metabolites between the samples from the three time points.

Multivariate statistical methods, partial least square discriminant analysis (PLS-DA), and orthogonal projections to latent structures discriminant analysis (OPLS-DA) were further applied to identify differential metabolites between the three groups of samples. Differential metabolites were identified with VIP ≥ 1 from OPLS-DA and *p*-value < 0.05 (see Supplementary Tables S1–S4). As shown in Figure 3, 95 metabolites were upregulated and 88 downregulated in the rehydration process (S2 vs. S1). In the subsequent drying stage (S3 vs. S2), only seven metabolites continued to increase, but 123 began to decrease. A comparation between the drying and initial stages (S3 vs. S1) found that 33 metabolites were upregulated and 137 downregulated. These changes implied that the metabolite biosynthesis mainly took place during the rehydration process, and the degradation or possible conversion mainly occurred during the dehydration process. 

**Figure 4:**
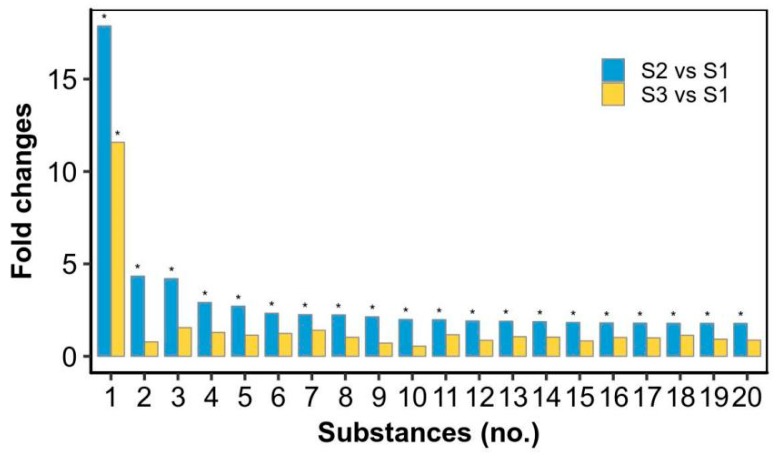
The top 20 significantly differential metabolites relatively upregulated in the rewetting stage and then reduced in the drying stage. * significant difference (*p*-value < 0.05), as compared to the S1 stage. 1—Ethenodeoxyadenosine; 2—TG(12:0/12:0/20:2(11Z,14Z))[iso3]; 3—3-Hexadecanoyloleanolic acid; 4—Hericenone E; 5—PA(12:0/15:1(9Z)); 6—Bacteriohopane-32,33,34-triol-35-carbamate; 7—Norselic acid E; 8—PG(18:4(6Z,9Z,12Z,15Z)/15:1(9Z)); 9—PE(17:0/20:4(5Z,8Z,11Z,14Z)); 10—PG(13:0/20:3(8Z,11Z,14Z)); 11—PS(O-16:0/18:3(9Z,12Z,15Z)); 12—PE(22:6(4Z,7Z,10Z,13Z,16Z,19Z)/20:4(5Z,8Z,11Z,14Z))[U]; 13—PS(O-16:0/18:4(6Z,9Z,12Z,15Z)); 14—MIPC(t18:0/22:0(2OH)); 15—PE(18:0/20:4(5Z,8Z,10E,14Z)(12OH[S])); 16—SQDG(16:0/16:0); 17—PS(12:0/21:0); 18—Mucronine A; 19—PE(16:0/22:6(4Z,7Z,10Z,13Z,16Z,19Z)); 20—PI(20:2(11Z,14Z)/22:4(7Z,10Z,13Z,16Z)).

The top 20 metabolites that were upregulated in the rehydration process and then reduced in the drying stage were summarized (Figure 4; Supplementary Tables S1–S4). As shown in Figure 4, 19 of the 20 metabolites were significantly increased at the S2 stage and reduced to the initial level at the S3 stage except ethenodeoxyadenosine (No. 1 substance). Ethenodeoxyadenosine has been shown to be a biomarker for lipid peroxidation-induced DNA damage in human cancer [18,19,20]. Lipid peroxidation produces reactive biomolecules such as trans-4-hydroxy-2-nonenal and malondialdehyde; the former can be further oxidized by hydrogen peroxide or fatty acid hydroperoxides to form its epoxide intermediate, which can attack nucleic acids to form the DNA adduct ethenodeoxyadenosine [18]. Ethenodeoxyadenosine showed an ~18 fold upregulation in the rehydration process and then reduced to an ~12 fold upregulation at the drying point. It was reported that the superoxide anion level was much higher (~4 fold) after 4 h of rehydration as compared to that after 48 h of dehydration, and the superoxide dismutase, catalase, and peroxidase were all higher at the former condition [13]. The activities of three antioxidant enzymes were also reported to be increased during a 25-h rehydration, but their protein abundances were somewhat decreased [14]. The oxidative damage of DNA occurs in cells as a consequence of normal aerobic metabolism and by environmental stress-triggered free radicals [21]. It was mentioned that when desiccated, genomic DNA was protected from oxidative damage in *Nostoc commune* [22]. Thus, we postulate that there exists a beneficial dynamic oxidation in the DNA level during the rehydration–dehydration process of *N. flagelliforme*, as indicated by the biomarker ethenodeoxyadenosine. The remaining 19 substances are generally related to lipid metabolism or possible cell signaling, according to the PubChem and HMDB databases. For example, 3-hexadecanoyloleanolic acid is found in fruits and belongs to the class of organic compounds known as triterpenoids; hericenone E is found in mushrooms and is a derivative of lineolic acid. An alteration in the lipid content of membrane is recognized to be of major importance for organisms in response to environmental stresses [23,24]. *N. flagelliforme* samples contain a high proportion of unsaturated fatty acids in membrane lipids [25]. Therefore, the rapid reduction of these substances in the drying stage implied an important intermediate or transitional role during the rehydration–dehydration cycle.

**Figure 5:**
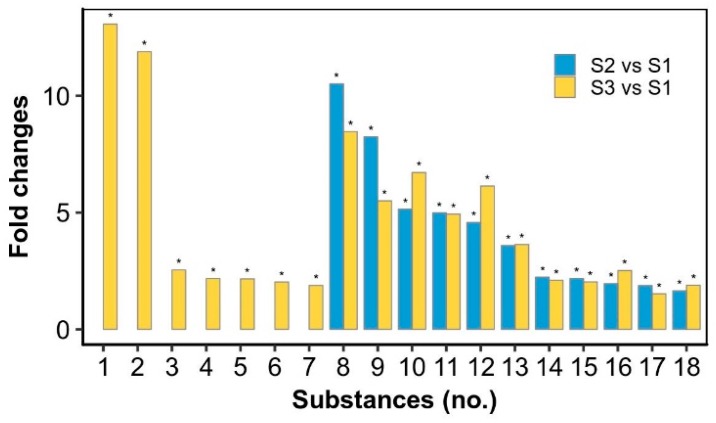
The significantly differential metabolites relatively upregulated in the drying stage or both the rewetting and drying stages. * significant difference (*p*-value < 0.05), as compared to the S1 stage. 1—2-Ethylacrylylcarnitine; 2—PC(O-12:0/O-1:0); 3—Dihydroskullcap flavone I; 4—Neuraminic acid; 5—(E)-Resveratrol 3-glucoside 4’-sulfate; 6—Echinenone/ (Myxoxanthin); 7—PE-Cer(d14:2(4E,6E)/24:1(15Z)(2OH)); 8—(S)-2,3-Dihydro-3,5-dihydroxy-2-oxo-3-indoleacetic acid 5-[glucosyl-(1->4)-b-D-glucoside]; 9—Fenothiocarb; 10—6-Methoxy-9H-carbazole-3-carboxaldehyde; 11—Levomethadyl acetate; 12—3-Indolebutyric acid; 13—CerP(d18:1/12:0); 14—PI(O-16:0/12:0); 15—PC(15:0/19:3(9Z,12Z,15Z))[U]; 16—Taxiphyllin; 17—PG(16:0/22:5(4Z,7Z,10Z,13Z,16Z)); 18—Phosphohydroxypyruvic acid.

We further summarized the top 18 metabolites that accumulated in the drying stage or did not obviously reduce in this process (Figure 5). These metabolites are related to diverse metabolic pathways, such as carbohydrate, lipid and amino acid metabolism, which might function during the dehydration process or in the desiccated state. 2-Ethylacryloylcarnitine was upregulated ~13 fold at the drying point. It is an *O*-acylcarnitine, an ammonium betaine, and a carboxylic ester. Betaine serves as an organic osmolyte and protects cells against osmotic, drought, and temperature stresses [26]. Thus, 2-ethylacryloylcarnitine might act as a novel stress biomarker. Neuraminic acid is a 9-carbon monosaccharide, a derivative of a ketononose, which was upregulated ~2 fold at the drying point. Many of its derivatives are found in glycoproteins and gangliosides, and the *N*- or *O*-substituted derivatives of neuraminic acid are collectively known as sialic acids with antiviral and antibacterial defensive functions [27,28]. Echinenone/Myxoxanthin, which was ~2 fold upregulated at the drying point, is a xanthophyll synthesized from β-carotene by the enzyme beta-carotene ketolase (CrtO) [29,30]. It can confer oxidative and desiccation stress tolerances in cells [31]. CrtO protein was decreased in the rehydration process [14] but increased in the dehydration (unpublished data) process. The potential 3-indoleacetic acid derivative (No. 8 substance) and 3-indolebutyric acid (No. 12 substance) were both highly increased in the rehydration and drying processes. Both 3-indoleacetic acid and 3-indolebutyric acid are plant hormones in the auxin family; the latter works as a storage sink for the former in plants [32]. It was reported that 3-indoleacetic acid promoted the growth of *N. flagelliforme* [33]. Both hormones were also reported to increase the activities of antioxidative enzymes in plants [34]. Fenothiocarb has been reported to be a water-soluble pesticide [35]. Taxiphyllin, a tyrosine-derived cyanogenic glycoside [36], has toxicity against *Artemia salina* and antibacterial activity. Levomethadyl acetate belongs to the class of organic compounds known as diphenylmethanes, which have therapeutic potential [37]. The synthesis of fenothiocarb, taxiphyllin and levomethadyl acetate might be required to resist other microorganisms or worms in the environment since *N. flagelliforme* maintains a free-living colony form, unlike soil biological crust. Phosphohydroxypyruvic acid was slightly upregulated in the rehydration and drying processes, which is a product of two enzymes, phosphoglycerate dehydrogenase and phosphoserine transaminase, in glycine and serine metabolism [38]. Phosphoglycerate dehydrogenases were detected to be increased in the rehydration process [14]. Taken together, the significant increases of these representative metabolites suggested potential diverse functions necessary for resistance to environmental abiotic or biotic stresses.

In addition, several metabolites showed changes from scratch or from existence to absence (Table 1). They may have been involved in more complex metabolic processes, either de novo synthesis or converted from other metabolites. These metabolites are also supposed to be important for environmental adaptation. For example, it was reported that 4-amino-o-cresol significantly inhibited soil urease activity [39]; (2’S)-Deoxymyxol 2’-(2,4-di-*O*-methyl-α-l-fucoside), as a domyxoxanthophyll derivative, may function as a photoprotectant [9]. They also serve as potential biomarkers to indicate the physiological states of *N. flagelliforme*.

### 2.4. Metabolic Pathway Analysis

**Figure 6:**
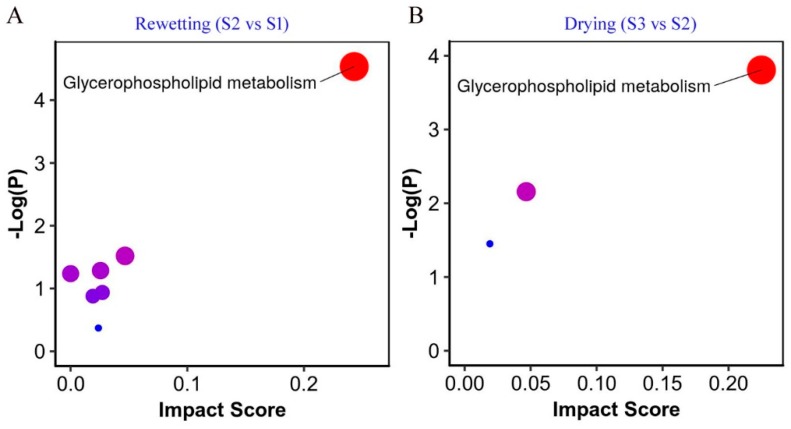
The metabolic pathway analysis of *N. flagelliforme* in response to the rewetting (**A**) and drying (**B**) processes. The impacted pathways are shown as circles. The size of the circle corresponds to the pathway impact score, and their colors are based on *p*-value.

Next, pathway enrichment analysis was performed by the MetaboAnalyst tool [40], with the aim to elucidate whether these differential metabolites were linked to specific biological processes (Figure 6). The glycerophospholipid metabolism was dramatically modulated during both the rewetting and drying processes, seemingly more prominent during the latter. Glycerophospholipid metabolism was also reported to be enhanced or favored during dehydration in other desiccation-tolerant species [41,42,43]. The maintenance of membrane integrity in anhydrobiotic organisms represents a central mechanism of desiccation [44]. The sugar trehalose has been well recognized to be crucial for inhibiting membrane fusion and maintaining the fluidity of membrane lipids during drying or in the absence of water [44]. This pathway analysis further emphasized the key of the timely membrane phase transition during the rehydration–dehydration cycle. Thus, the dynamic changes of phospholipids should be a critical aspect of *N. flagelliforme* adapting to the environmental moisture regime.

## 3. Material and Methods

### 3.1. Organisms, Culture Conditions, Sampling and Treatments

*N. flagelliforme* samples were collected from the xeric steppes in Zhongwei, Ninxia Province, China. Filamentous air-dried samples (water content 10.2%) were washed three times with deionized water, surface-sterilized by 75% ethanol for 30 s, and washed again with sterilized water two times. These treatments were conducted at 4 °C in darkness. This ethanol sterilization could eliminate most or all epiphytic microorganisms on the surface of *N. flagelliforme* filaments. Partial surface-disinfected samples were immediately collected and stored at −80 °C (Sample 1). Others were further subjected to rewetting and subsequent drying treatments. During both processes, relative weight changes (folds) were calculated by comparing the wet weights of water-absorbed samples with the initial dry weights. A sensitive PSII activity parameter Fv/Fm was also detected by a Plant Efficiency Analyzer as previously described [45].

For rehydration, samples were immersed in BG11_0_ solution at 25 °C and a continuous illumination of 40 µmol photons m^−2^·s^−1^ for physiological recovery. As often observed, a fully physiological recovery, in terms of Fv/Fm, was achieved at 16–20 h. At 20 h, partially rehydrated samples were collected and immediately stored at −80 °C (Sample 2). The remaining rehydrated samples were subjected to a dehydration treatment at the same temperature and light intensity conditions. After removing surface water with filter paper, the samples were naturally dried in petri dishes with perforated lids. The environmental relative humidity was around 42%. The samples with a relative water loss of 50% and a significant reduction of Fv/Fm (as compared to those in the samples at 20 h) were collected and stored at −80 °C (Sample 3). Eight replicates were performed in each experiment. The three groups of samples were freeze-dried in a freeze dryer (CoolSafe^TM^, SCANVAC, LabGene, Denmark) and sent with dry ice to the Metabolome Ingenuity Technology Bio-company (Shanghai, China) for metabolomic analysis.

### 3.2. Metabolite Extraction

Each sample of 200 mg was weighted and mixed with 1 mL methanol (HPLC grade, Merck, Germany) containing 5 μg/mL 2-chloro-l-phenylalanine (Sigma-Aldrich, St. Louis, MO, USA) as an internal standard. The mixture was homogenized at 60 Hz for 2 min and vibrated in a vortex mixer for 2 min. The mixture was then centrifuged at 4 °C by 13,400 *g* for 10 min, and the supernatant of 200 μL was filtered through a syringe filter (0.22 μm) and transferred to a sampling vial for LC-MS analysis. An in-house QC was prepared by mixing equal amounts of the tested samples.

### 3.3. LC-MS Analysis

LC-MS analysis was performed by an Agilent 1290 Infinity II UHPLC system (Santa Clara, CA, USA) coupled to an Agilent 6545 UHD and an Accurate-Mass Q-TOF/MS. A 2 μL aliquot of the filtrate was injected into a Waters XSelect HSS T3 column (100 × 2.1 mm, 2.5 µm, Waters, Manchester, UK) held at 25 °C. The column was eluted with gradient elution using mobile phases A and B. Mobile phase A is an aqueous solution containing 0.1% formic acid (Sigma-Aldrich). Mobile phase B is an acetonitrile solution (HPLC grade, Merck, Germany) containing 0.1% formic acid. The optimized gradient elution condition was: 0–2 min, 5% B; 2–10 min, 5–95% B; 10–15min, 95% B; 15–18 min, 95–5% B. The post time was set as 3 min for system balance.

Mass spectrometry was operated in both positive and negative ionization modes. The parameters optimized were as follows: capillary voltage, 4 kV in positive mode and 3.5 kV in negative mode; drying gas flow, 10 L/min; gas temperature, 325 °C; nebulizer pressure, 20 psig; fragmentor voltage, 120 V; skimmer voltage, 45 V. The mass data were collected at the range of *m*/*z* 100–1700. 

### 3.4. Metabolomic Analysis

The raw LC-MS data were converted to the common format (mz.data) by Agilent Masshunter Qualitative Analysis B.08.00 software (Agilent Technologies). In the R software platform, the XCMS program was used for peak identification, retention time correction, and automatic integration. The data were then subjected to internal standard and weight normalization, achieving visualization matrices including sample name, *m*/*z*-RT pair and peak area. After editing, the data matrices were imported into SIMCA-P 13.0 (Umetrics, Umea, Sweden) for mean-centering, Pareto scaling and then multivariate analyses, including PCA, PLS-DA, and OPLS-DA [1,46]. 

### 3.5. Identification of Differential Metabolites

Differential metabolites between different groups were identified by the VIP (Variable Importance in the Projection) value of the OPLS-DA model (VIP ≥ 1) and the independent sample *t*-test (*p*-value < 0.05). The qualitation of the differential metabolites was performed by searching in the Metlin online database for an accurate molecular weight comparison. The adduct manners were: [M + H]^+^ and [M + Na]^+^ in positive mode, [M – H]^−^ and [M + FA – H]^−^ in negative mode; the mass error value was 20 PPM. Peptides, drugs, and toxicants were removed from the compounds searched.

## 4. Conclusions

*N. flagelliforme* is an edible cyanobacterium with herbal value inhabiting xeric environments. Small molecular metabolites and their dynamic changes during the rehydration–dehydration cycle were revealed by untargeted metabolomics in this study. This is the first report interpreting this issue in *N. flagelliforme*. Among the metabolites identified, some may serve as ideal biomarkers that are indicative of physiological changes of *N. flagelliforme*, such as those mainly synthesized in the rehydration process and then reduced in the drying process. In addition, those induced during the rehydration or drying process and then existing in dormant samples might show their importance for desiccation resistance. The interpretation of these metabolites suggests that they serve as diverse functions in the environmental adaption of *N. flagelliforme*, either to abiotic or biotic stresses. In addition, we found obvious lipid metabolism dynamics during the rehydration–dehydration process. It implies that the rapid phase transition of membrane is a critical process for *N. flagelliforme* under periodic desiccation. This further enriches our understanding on its environmental adaption except for the well-known pigmented exopolysaccharide sheath. However, it should be noted that the current metabolomic analysis only revealed the moisture-associated metabolite changes. In native environments, other abiotic stressors, such as ultraviolet radiation, may together result in more complex metabolic profiles. It is also noteworthy that some metabolites have antibacterial activity or therapeutic value. Therefore, this study also provides cultural clues to biotechnologically achieve the efficient production of these metabolites from *N. flagelliforme* in future commercial markets.

## Figures and Tables

**Table 1:** The metabolites that showed changes from scratch or from existence to absence during the rewetting–drying process.

Substances	Formula	S1	S2	S3
4-Amino-o-cresol	C7H9NO	-	+	++
(2’S)-Deoxymyxol 2’-(2,4-di-O-methyl-α-l-fucoside)	C48H70O6	-	+	+
PE(15:1(9Z)/22:6(4Z,7Z,10Z,13Z,16Z,19Z))	C42H70NO8P	-	+	+
MGDG(18:5(3Z,6Z,9Z,12Z,15Z)/18:5(3Z,6Z,9Z,12Z,15Z))	C45H66O10	-	+	+
Asparaginyl-Threonine	C7H9NO	-	-	+
Tropine	C8H15NO	-	-	+
Coronene	C24H12	-	-	+
PC(18:0/19:3(9Z,12Z,15Z))[U]	C45H84NO8P	++	+	-
PS(19:1(9Z)/22:6(4Z,7Z,10Z,13Z,16Z,19Z))	C47H78NO10P	+	-	-
DG(13:0/18:3(9Z,12Z,15Z)/0:0)[iso2]	C34H60O5	+	-	-
PC(18:4(6Z,9Z,12Z,15Z)/20:5(5Z,8Z,11Z,14Z,17Z))	C46H74NO8P	+	-	-
9-hydroperoxy-12,13-epoxy-10-octadecenoic acid	C18H32O5	+	-	-
CDP-DG(18:0/18:0)	C48H89N3O15P2	+	-	-
4-Ketonostoxanthin 3-sulfate	C40H53NaO8S	+	-	-
TG(12:0/12:0/18:1(9Z))[iso3]	C45H84O6	+	-	-

-, absence; +, low amount; ++, high amount.

## Supplementary Materials

The following are available online at https://www.mdpi.com/1660-3397/17/5/298/s1: Table S1. The metabolites detected in both positive and negative modes; Table S2. The list of differential metabolites between S2 and S1; Table S3. The list of differential metabolites between S3 and S2; Table S4. The list of differential metabolites between S3 and S1.
